# One-Pot Synthesis of Functionalised rGO/AgNPs Hybrids as Pigments for Highly Conductive Printing Inks

**DOI:** 10.3390/nano14100859

**Published:** 2024-05-15

**Authors:** Vassiliki Belessi, Apostolos Koutsioukis, Dimitra Giasafaki, Theodora Philippakopoulou, Vassiliki Panagiotopoulou, Christina Mitzithra, Sotiria Kripotou, Georgios Manolis, Theodore Steriotis, Georgia Charalambopoulou, Vasilios Georgakilas

**Affiliations:** 1 Department of Graphic Design and Visual Communication, Graphic Arts Technology Study Direction, University of West Attica, Egaleo, 12243 Athens, Greece; theofil@uniwa.gr; 2 Laboratory of Electronic Devices and Materials, Department of Electrical and Electronic Engineering, University of West Attica, Egaleo, 12244 Athens, Greece; skrypotou@uniwa.gr; 3 Department of Materials Science, University of Patras, 26504 Rio, Greece; apostoliphone@gmail.com; 4 National Centre for Scientific Research “Demokritos”, Agia Paraskevi, 15341 Athens, Greece; d.giasafaki@inn.demokritos.gr (D.G.); mitzithra.christina@gmail.com (C.M.); g.manolis@inn.demokritos.gr (G.M.); gchar@ipta.demokritos.gr (G.C.); 5 Druckfarben Group, Megaridos Ave, Thesi Kallistiri, 19300 Aspropirgos, Greece; vpanagiotopoulou@druckfarbengroup.com

**Keywords:** graphene, conductive inks, reduced graphene oxide, gravure, flexography, screen printing

## Abstract

This work provides a method for the development of conductive water-based printing inks for gravure, flexography and screen-printing incorporating commercial resins that are already used in the printing industry. The development of the respective conductive materials/pigments is based on the simultaneous (in one step) reduction of silver salts and graphene oxide in the presence of 2,5-diaminobenzenesulfonic acid that is used for the first time as the common in-situ reducing agent for these two reactions. The presence of aminophenylsulfonic derivatives is essential for the reduction procedure and in parallel leads to the enrichment of the graphene surface with aminophenylsulfonic groups that provide a high hydrophilicity to the final materials/pigments.

## 1. Introduction

Graphene oxide (GO) is a hydrophilic derivative of graphene that is typically produced by the strong oxidation of graphite [1]. The hydrophilic character of GO is due to the introduction of oxygen groups such as carboxylates, hydroxylates and epoxy groups on the graphene surface during the oxidation process [1,2]. GO is nonconductive in contrast with pristine graphene, because of the decreased aromaticity that is also induced by the oxidation. Nevertheless, the reduction of GO leads to conductive derivatives, known as reduced graphene oxide (rGO), whose conductivity depends on the reduction method used [3].

The high conductivity of graphene derivatives such as rGO along with the fact that it can be produced very easily on a large scale from graphite, an abundant raw material, makes it particularly suitable for the development of metal-free, eco-friendly, and inexpensive conductive inks [4,5]. Recently, we have shown that the treatment of GO with 2,4- and 2,5-diaminobenzenesulfonic acid (2,4-DBSA, 2,5-DBSA) leads to an effective reduction and a partial functionalization of GO with aryl sulfonic groups [6]. Also, Fukaya et.al. [7] studied the adsorption and reduction reactions between GO and 2,4-DBSA, and 2,5-DBSA through experimental and numerical analyses and showed that 2,5-DBSA possesses a stronger reducing ability towards GO than 2,4-DBSA. The as produced functionalized rGO (*f*-rGO) is highly reduced and simultaneously hydrophilic and is, therefore, considered ideal for use as a conductive functional pigment for the development of water-based conductive inks for gravure [6], flexo [8] and ink-jet printing [9,10,11,12], paving the way for its utilization in many application areas.

Indeed, conductive inks are widely used for the fabrication of a broad range of devices for specialized applications, from sensors, integrated circuits, wearable electronics, radiofrequency identification tags, to energy harvesting/storage systems [9,10,13,14,15,16,17,18,19]. For example, Barmpakos et al. [9,10] showed that commercial graphene and *f*-rGO [6] can perfectly act as temperature sensors as well as heaters with a stable response and durability to thermal stressing for ink-jet printed devices. Specifically, it has been reported that in the range of −40–100 °C, the Thermal Coefficient of Resistance was −1.05 × 10^−3^ °C^−1^ and −3.86 × 10^−3^ °C^−1^ for graphene and *f*-rGO, respectively, featuring the superiority of *f*-rGO behavior. Furthermore, *f*-rGO can reach a maximum temperature of 100 °C with 250 mW, while graphene requires 28% more power (320 mW) to reach the same temperature on a 125 μm-thick polyimide substrate. Recently, a sustainable high-conductive (3 × 10^4^ Sm^−1^) screen-printing graphene ink was used to develop a battery-free wireless UHF RFID temperature sensing tag antenna on common paper [16]. It was observed that the read range was nearly half a meter. Thus, the development of conductive materials suitable for conductive inks is a research field of strong interest because it can contribute decisively to the growth of the global printed electronics market.

Hybrid inks which are based on graphene derivatives containing metal nanoparticles such as silver (AgNPs) have been reported as an alternative approach for improving the electrical properties of conductive inks [13,20,21,22,23,24,25,26]. These systems frequently present silver inhomogeneity issues [20,27] since producing stable AgNPs in water-based inks is challenging or requires time-consuming and complex methods [20,28,29]. However, the *f*-rGO derivative [6] that has been successfully used before for the development of hydrophilic and highly conductive composites such as *f*-rGO/Ag nanowires [30] and *f*-rGO/Ag nanoparticles (*f*-rGO/AgNPs) [31], favors the preparation of stable graphene/Ag conductive inks. Specifically, it has been shown that such hybrid carbon-based materials remain water-dispersible without the need for any additive, and of low sheet resistance without the necessity of sintering or annealing. Especially, in the case of *f*-rGO/AgNPs [31], it was found that the carbon substrate (e.g., *f*-rGO or few-layered graphene nanosheets) had a crucial role in determining the morphology and the properties of the final hybrid, while AgNPs are much better dispersed in *f*-rGO than in the few-layered graphene nanosheets. Also, it was found that the homogeneous dispersion of AgNPs on the *f*-rGO creates conductive interconnections between the graphene nanosheets, improving the electrical conductivity of the hybrid.

The in situ reduction of Ag ions is the simplest and most efficient method for the deposition of nanoparticles on graphene [29,32,33,34,35]. It can be performed with various reducing agents such as sodium borohydride (NaBH_4_), potassium hydroxide (KOH), and ascorbic acid [20,34,35,36,37,38,39]; however, it also requires two steps: the reduction of silver to form nanoparticles and the reduction of GO to rGO. The successful preparation of conductive graphene/silver nanoparticle hybrids in one step [20] is of great importance for the scalability of graphene-based technologies and their transfer from the laboratory to the industrial production scale. So far, this has been achieved by using mainly formaldehyde, ascorbic acid or tannic acid; however, the reducing action is relatively weak [20,40]. In some other cases, the simultaneous application of microwaves or another energy source has been necessary [40]. The electrical conductivities of these hybrid materials are in the range of 0.3–2.0 × 10^3^ S cm^−1^; however, such values are usually achieved by heat treatment at temperatures between 100 and 400 °C [21,26,40,41] which not only affects the energy demands and the overall cost of the process but also excludes the use of heat-sensitive substrates. Clearly, there is still a need for improved routes for the synthesis of appropriate graphene-based hybrid materials and their efficient incorporation in the industrial production of conductive inks, and particularly of water-based conductive inks.

In this work, we present an easy, eco-friendly, cost-effective, one-pot synthesis of a conductive hybrid combining *f*-rGO and Ag NPs. The treatment of a mixture of GO and Ag ions in water with 2,5-diaminobenzenesulfonic acid (2,5-DBSA) led to the simultaneous reduction of GO and Ag ions and the functionalization of the produced rGO with aryl sulfonic groups. It should be noted that to the best of our knowledge, this is the first time 2,5-DBSA is used as the common reducing agent driving these two reactions. Ag NPs were deposited on the *f*-rGO surface leading to a highly hydrophilic and conductive hybrid. We have considered various reducing agents to assess the efficiency of 2,5-DBSA, for example, phenyl diamines, aliphatic dia-, tria- and tetramines, monoamino and diaminobenzenesulfonic acids, benzenesulfonic acids, etc. It was shown that the three isomers of diaminobenzenesulfonic acids (2,4-, 2,5-, and 3,4-DBSA) and/or combinations of them at any ratio had significantly superior behavior concerning the hydrophilicity and the conductivity of the derived hybrids [6].

The hydrophilic character of the conductive hybrid was found to improve its incorporation into commercial water-based resins that are necessary components for the development of printing inks, without requiring the addition of surfactants or other hydrophilic polymers, for the stabilization of graphene. Finally, it has been demonstrated that the as-prepared inks are very well suited for various mature printing methods such as gravure, flexography and screen printing as required for the development of printed electronics.

## 2. Experimental Section

### 2.1. Materials and Methods

Graphite (powder, synthetic, particle size < 20 μm), 2,5-diaminobenzenesulfonic acid (tech., 90%) (2,5-DBSA) and silver nitrate were purchased from Aldrich. Sulfuric acid (95–97%) and potassium chlorate (purum > 99.0%) were purchased from Merck and nitric acid (65%) from Riedel-de Haen. All the solvents were of analytical grade and were used as received. The resins Joncryl 1685 (acrylic emulsion), Joncryl 8052 (acrylic film-forming emulsion), Joncryl MB90 (environmentally advanced styrene-acrylic emulsion, biomass balance product and non-film-forming) were received from BASF HELLAS. The solid content of the resins was estimated to be 43.5 (Joncryl 1685), 46.5 (Joncryl 8052) and 44 wt% (Joncryl MB90).

### 2.2. Preparation of Graphene Oxide (GO)

Graphite oxide was synthesized by a modified Staudenmaier method [42,43]; 2 g of powdered graphite was added to an ice-cooled flask containing a mixture of concentrated sulfuric acid (80 mL) and nitric acid (40 mL). Potassium chlorate (40 g) was slowly added to the mixture while stirring and cooling. The reactions were quenched after 18 h by pouring the mixture into distilled water and the product was isolated by centrifugation (9000 rpm) and washed with water several times until the pH of the supernatant was almost neutral. Finally, the sample was dried at room temperature.

### 2.3. One Pot Synthesis of f-rGO/AgNP Hybrid

An amount of 75 mg of GO was dispersed in 75 mL of deionized water and stirred for 24 h. The dispersion of the GO was ultrasonicated for 30 min using a Branson 3800 bath sonicator (110 W, 40 kHz). Then, an appropriate amount of AgNO_3_ was added to the GO suspension in order to obtain hybrids with 5.0–15.7 wt% of Ag and the mixture remained overnight under stirring. Hereupon, 150 mg of 2,5-diaminobenzenesulfonic acid (2,5-DBSA) were added to the dispersion and the mixture was refluxed for 2 h under stirring. After cooling the mixture to room temperature, it was vacuum filtered through Nylon membrane filters with 0.45 μm pore size (Whatman). The obtained solid was washed with water, ethanol and acetone. The product was further purified using a dialysis membrane (3,5 kDA) and dried at room temperature prior to its structural characterization with various methods. The same experimental procedure was also successfully used for m_2,5-DBSA_/m_GO_ ratios ranging between 2 and 6.

### 2.4. Preparation of Conductive Inks

The procedure described above was properly adapted in order to collect the amount of the hybrid material required to produce a sufficient batch of ink. Briefly, 400 mg of GO were dispersed in 300 mL of deionized water, stirred for 24 h and the GO dispersion was ultrasonicated for 30 min. Then, 58 mg of AgNO_3_ were added to the GO suspension to obtain an 8.5 wt% Ag loading. In a next step, 800 mg of 2,5-DBSA were added to the dispersion, the mixture was refluxed for 2 h, washed, vacuum filtered and dried as previously described. The mass of the produced *f*-rGO/AgNPs hybrid was 320 mg (reaction yield 80% per batch).

The hydrophilic sample *f*-rGO/AgNPs that contains 8.5% wt. AgNPs was selected as the most suitable for the preparation of inks considering energetic, operational and environmental parameters (such as the production energy cost, the conductivity/electrical properties, and the extent of the discharge of metal particles waste into the environment) and will hereafter be designated as “*f*-rGO/AgNPs pigment”.

The resin system that was used for the inks was made by mixing the following emulsions: Joncryl 1685, Joncryl 8052 and Joncryl MB90 at a 60:20:20 ratio. More specifically, two printing inks were formulated: (a) one for gravure and flexography with total solids pigment/resin ratio 55/45 and (b) a second one for screen printing with total solids pigment/resin ratio 70/30 (Table 1). In the first case, 2.2 g of the *f*-rGO/AgNPs pigment was mixed with 4.0 g of the water-based resins mixture; in total, the water volume required to prepare an ink quantity sufficient for gravure and flexography printing (at a solids pigment/resin ratio of 55/45) was 12.0 mL and the respective ink is labeled as Gravure-Flexo Ink P55/R45. In the latter case, 2.1 g of the *f*-rGO/AgNPs pigment was combined with 2.0 g of the resin mixture, while the water volume for preparing enough ink for screen printing at a solid’s pigment/resin ratio of 70/30 was 6.0 mL. That ink is labeled as Screen Ink P70/R30.

### 2.5. Characterization of Materials

X-ray powder diffraction (XRD) patterns were collected on a D-500 Siemens diffractometer using Cu Kα radiation (λ = 1.5418 Å).

Thermogravimetric analysis (TGA) was performed on a SETARAM SETSYS Evolution Analyser in the range of 25–1100 °C, using an alumina crucible, and a heating rate of 10 °C/min under airflow (16 mL/min).

Transmission electron microscopy (TEM) images were obtained using an FEI Talos F200i S/TEM field emission gun scanning electron microscope, operating at 200 kV.

Raman spectra were measured in a backscattering configuration at room temperature, in the 300−3700 cm^−1^ range by a dispersive Renishaw in-Via Reflex spectrometer. Excitation was performed by a solid-state laser emitting at a wavelength of 514 nm. The excited spot of ~5 μm in diameter on the sample’s surface was achieved by a 20× objective lens of a Leica DMLM microscope. In order to increase the statistics, a mapping at 5 × 5 spots has been performed on each sample surface. The power density, approximately 0.02 mW/μm^2^, was well below the damage threshold in order to avoid sample overheating.

The resistance (V/I) of the *f*-rGO/AgNPs (8.5% wt.) pigment, the *f*-rGO/AgNPs inks and the printed patterns were measured by a 4-point probe system (Lucas Labs, Lucas SignatoneCorp., Gilroy, CA, USA) and a Keithley 2400 Source Meter (Keithley Instruments, Cleveland, OH, USA). Then, the sheet resistance (R_s_) values were calculated according to our previous work [6,32,44,45,46].

Optical absorption (OA) spectra in the UV-Vis spectral region were recorded on a Shimadzu 1650 spectrophotometer in the range 200–800 nm, at a sampling step of 0.5 nm with 1.5 nm slits, using a combination of halogen and deuterium lamps as sources.

### 2.6. Characterization of the Resins Mixture and Ink

#### 2.6.1. Drawdawn Test

To design a resins mixture suitable for water-based inks for gravure, flexo and screen printing, the three commercial emulsions (Joncryl 1685, Joncryl 8052 and Joncryl MB90) were initially mixed at various weight ratios (0:1:0, 0:0:1, 1:0:0, 1.3:1:1, 2:1:1, 3:1:1, 4.7:1:1, 8:1:1). Each of the three resin emulsions, as well as their mixtures, were applied on various types of papers: common or special such as Leneta 3NT-33 (RK Print Coat Instruments, Cambridgeshire, UK), uncoated or coated paper (CT 2846, IGT Testing Systems, Almere, The Netherlands) and photocopy paper. Also, for the best visual inspection of the applied resin film, an appropriate amount of copper phthalocyanine blue pigment was dissolved in the binder solution (11% *w*/*w*) and the resins were evaluated as regards their adhesion on a paper substrate (ASTM-D3359-09e2, 25 Standard Test Methods for Measuring Adhesion by Tape Test, ASTM International), scratch resistance, water rub resistance, heat resistance and printability. For comparison, reference inks were also prepared by combining the resins with an aqueous carbon black dispersion (also 11% *w*/*w*). To simulate how a resin or the resins mixture or an ink appears on a specific substrate and choose the most appropriate types of paper, the wire-wound K Bars were numbered 0, 1, 2 and 4 (K Hand Coater, RK Print Coat Instruments) giving an intermediate wet film thickness of 4, 6, 12 and 40 μm, respectively.

#### 2.6.2. Heat Resistance

The resin emulsions were tested for heat resistance using the Brugger HSGCC Heat sealing machine (80 °C, 780 N, 0.1 s). In order not to damage the substrate, the test was carried out with the printed surface in contact with washed and unlacquered aluminum foil (10 μm thickness).

#### 2.6.3. Adhesion Test

The drawdown samples were used in order to evaluate the dry ink film adhesion force on a paper substrate (ASTM-D3359-09e2, 25 Standard Test Methods for Measuring Adhesion by Tape Test, ASTM International). Similar results were obtained using the printed patterns with the conductive ink. To perform the adhesion test, a pressure-sensitive tape was used (3M 610). The qualitative evaluation of the sample was based on visual inspection.

#### 2.6.4. Scratch Resistance

The STM 2011UK method was used to test the scratch resistance of the conductive inks and the resin binders on the substrate. The printed samples were placed horizontally and scratched by using a fingernail on the printed image.

#### 2.6.5. Water Rub Resistance

Water spots were applied on the printed surface for 1 min and then rubbed off. The result of re-dissolution was visually evaluated as the degree of color change before and after rubbing.

#### 2.6.6. Rheology

A rotational rheometer (Malvern Kinexus Pro+, Malvern Panalytical Ltd., Worcestershire, UK) was used for the rheological characterization of the flexo/gravure and screen-printing inks. For the measurements, a cone (40 mm diameter and 4° angle) and plate geometry were used. A shear strain rate ramp from 0.1 to 1000 s^−1^ was applied, while the temperature was kept constant at 25 °C.

#### 2.6.7. Gravure, Flexography and Screen Printing of the *f*-rGO/AgNPs Ink

For gravure printing, the printing tests were conducted using the IGT G1-5 printability tester (IGT Testing Systems) and the chromium-plated printing cylinder 402.226 (60, 80, 100, and 140 lines cm^−1^; screen angle 53; stylus angle 130 and cell volumes of 16, 11, 9, and 7 mL m^−2^). The printing force between the engraved disc and the substrate was 200 N and the printing speed was 0.6 m s^−1^.

For flexography, the printing tests were conducted using the IGT F1 printability tester (IGT Testing Systems) and various photopolymeric plates that offer compatibility with water-based inks (e.g., a. Cyrel DPN 67, DuPont, thickness 1.70 mm, 67 Shore A hardness, b. Cyrel DPR 45, DuPont, thickness 1.14 mm, 76 Shore A hardness and c. Torelief WF80DHX4, Toray, thickness 0.80 mm, 65 Shore D hardness). In all experiments, an anilox roller of 16 mL m^−2^ (402.413, IGT Testing Systems) was used, the anilox/printing force was 50 N, while the anilox/printing speed was studied in the range 0.2–1.5 m s^−1^ and a doctor blade pressure 6 N.

The screen-printing tests of the conductive inks were performed using a manual screen-printing machine (one color, one station Kunshan), an aluminum screen-printing frame with 90–40 mesh count (PE AM 90.48 PW, Saati) and a squeegee.

## 3. Results-Discussion

With the addition of 2,5-DBSA and heating under reflux, the two precursors (GO and Ag ions) were successfully reduced [47] and according to our previous results [6,30], rGO was functionalized by arylsulfonic groups and obtained a characteristic hydrophilic character. Similarly, Fukaya et al. [7] reported that the distance of the amino- and sulfo- groups in 2,5-DBSA (para-positions) is a determining factor for the strength of the electronic interaction between them and thus allows the significant reduction of GO by DBSA. Furthermore, the density functional theory calculations that the same authors performed indicated that 2,5-DBSA can have a stronger reduction effect than 2,4-DBSA (meta-positions).

**Figure 1:**
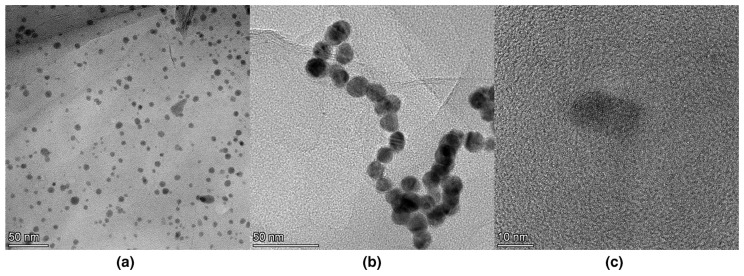
TEM images of the Ag nanoparticles deposited on *f*-rGO as a result of the one pot reduction of GO and Ag ions.

The morphology of the *f*-rGO/AgNPs hybrid material was studied by TEM. The respective images (Figure 1a–c) reveal the presence of spherical Ag nanoparticles with a diameter between 10 and 15 nm and a very narrow size distribution. The Ag nanoparticles were finely dispersed on the graphene surface (Figure 1a). In several places, Ag nanoparticles were aggregated forming rows as presented in Figure 1b.

### 3.1. Dispersibility and Stability of f-rGO/AgNPs (8.5% wt.)

**Figure 2:**
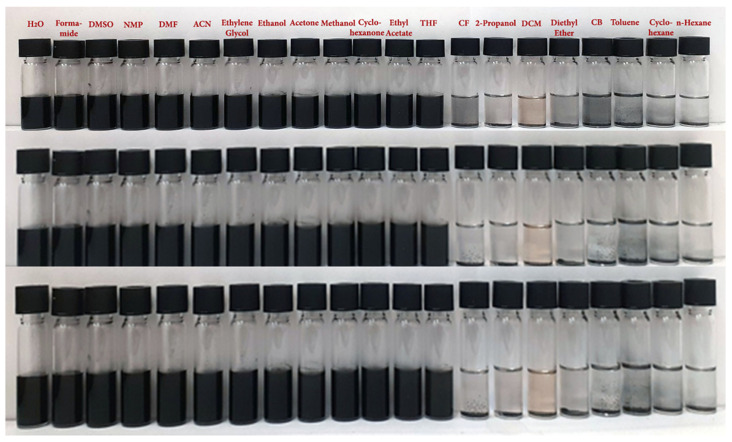
Dispersion of *f*-rGO/AgNPs pigment in a series of polar and non-polar solvents. The order of the solvents in each row follows the decrease in their polarity as represented by the polarity index. Top: immediately after sonication. Middle: after 48 h. Bottom: after 1 week.

Generally, GO is poorly dispersed in isopropanol, better in DMSO and ethanol and well in water or DMF [48,49,50]. On the other hand, the rGO prepared using thermal reduction or simple reducing agents such as NaBH_4_ is usually not dispersible in any solvent [48]. To study the dispersion in various solvents of the *f*-rGO/AgNPs (8.5% wt.) pigment that has been prepared in this work as described in paragraph 2.4, powder of the pigment was dispersed in 2 mL of a series of solvents in a concentration of 0.5 g L^−1^ (Figure 2). Specifically, the hybrid was dispersed in the polar solvents water, formamide, dimethyl sulfoxide (DMSO), 1-Methyl-2-pyrolidone (NMP), N,N-Dimethylformamide (DMF), acetonitrile (ACN), ethylene glycol, ethanol, acetone, methanol, cyclohexanone, ethyl acetate, tetrahydrofuran (THF), chloroform (CF), 2-propanol (IPA) and in the non-polar organic solvents dichloromethane (DCM), diethyl ether, chlorobenzene (CB), toluene, cyclohexane and n-hexane. It was found that the hybrid was dispersible in most polar solvents at least for one week as shown in Figure 2 and precipitated in non-polar organic solvents. Ethanol and water dispersions of the product at a concentration of up to 0.5 g L^−1^ were stable for more than four months (Figure 2). The pigment was also dispersed in water or ethanol at concentrations up to 30 g L^−1^, similar to our previous work [6,30], giving highly viscous homogeneous mixtures without adding surfactants or other additives for the stabilization of graphene sheets.

**Figure 3:**
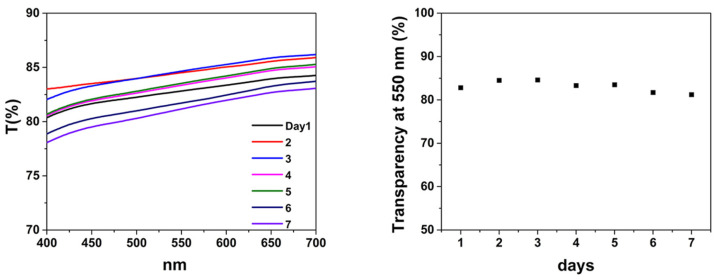
Stability test of the dispersion of *f*-rGO/AgNPs in water based on the transparency at 550 nm during one-week measurements.

The stability of the water or ethanol dispersion of *f*-rGO/AgNPs was further examined by UV-Vis, measuring its transmittance at 550 nm as a function of time. The transmittance of *f*-rGO/AgNPs in water remained almost stable during a period of seven days (Figure 3). Likewise, the stability of the ethanol dispersion of *f*-rGO/AgNPs was also examined by measuring its transmittance at 550 nm as a function of time, which again remained almost stable over a week.

**Figure 4:**
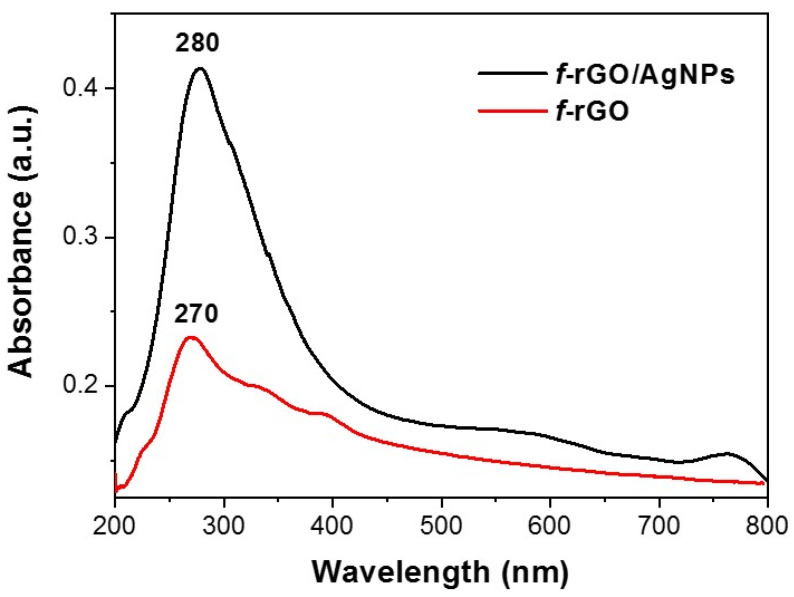
UV–Vis spectra of *f*-rGO and *f*-rGO/AgNPs.

The UV–Vis spectrum of *f*-rGO/AgNPs is presented in Figure 4 showing an absorption peak at 280, due to the π-π* transition of aromatic C=C bonds of the graphene component, and a broad peak between 500 nm and 600 nm. The corresponding absorption spectrum of graphene obtained after reduction without the presence of AgNPs showed a peak at 270 nm due to the π-π* transition and a broad shoulder at 350 nm due to the n-π* transitions of the remaining oxygen groups. The 10 nm red shift of the peak corresponding to the π-π* transition and the absence of any signal between 300 and 400 nm in the absorption spectrum of *f*-rGO/AgNPs indicate an extended reduction of GO in the presence of Ag. Also, the broad peak between 500 and 600 nm is attributed to the existence of Ag NPs on the surface of *f*-rGO due to the excitation of surface plasmon vibration.

### 3.2. XRD Analysis of f-rGO/AgNPs

**Figure 5:**
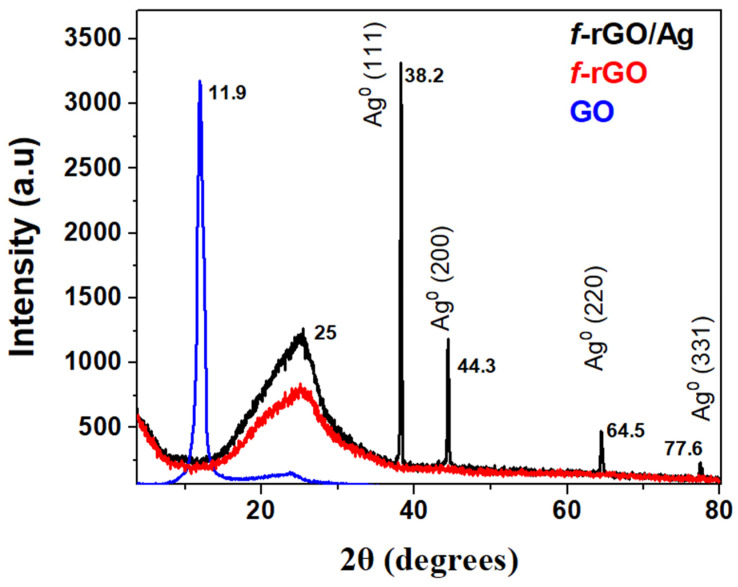
Powder XRD patterns of GO, *f*-rGO and *f*-rGO/AgNPs.

The XRD pattern of the *f*-rGO/AgNPs pigment compared to that of the starting GO and *f*-rGO is shown in Figure 5. The XRD pattern of GO shows a sharp peak at 11.9° corresponding to an interlayer distance of approximately 0.73 nm, due to the oxygen-containing functional groups of GO and water molecules that were normally entrapped between the GO nanosheets. This peak is absent from the *f*-rGO/AgNPs and *f*-rGO patterns and instead, a new broad peak appears at 25° corresponding to an interlayer distance of ~0.34 nm near the d_002_ spacing of graphite (0.335 nm), indicating the partial graphitic character of the *f*-rGO/AgNPs. Moreover, the XRD pattern of *f*-rGO/AgNPs contains all the characteristic peaks of Ag^0^ corresponding to the different crystallographic planes of phase-centered cubic Ag crystals, indicating the full reduction of both the Ag ions and the GO sheets.

### 3.3. TGA Measurements of f-rGO/AgNPs

**Figure 6:**
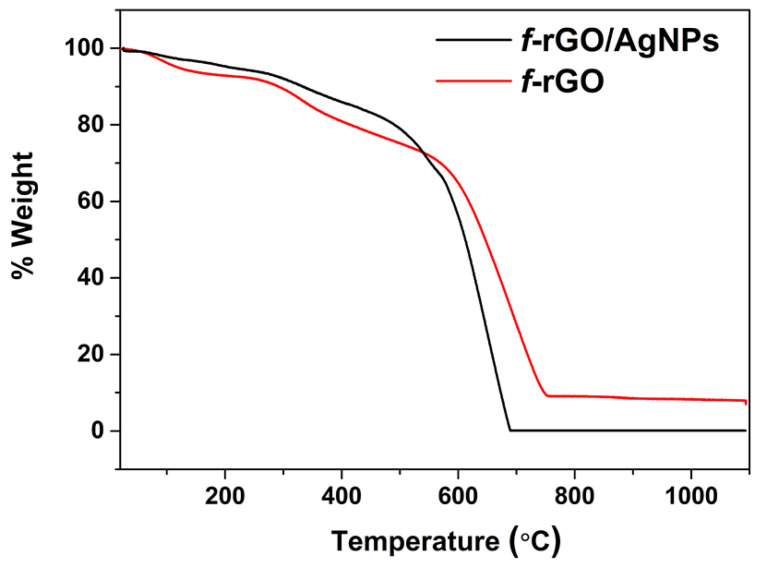
TGA thermograms of *f*-rGO and *f*-rGO/AgNPs in air.

The TGA diagram of the *f*-rGO/AgNPs hybrid showed the degradation of the graphene component between 600 and 750 °C. A residue unburnt material of 7% corresponds to the Ag loading in the hybrid (Figure 6). The thermogram also shows a weight loss step between 200 and 500 °C due to the removal of oxygen and especially aryl sulfonate groups from the graphene derivative. The similar degradation observed in the thermographs of *f*-rGO and *f*-rGO/AgNPs up to 600 °C indicates that the reduction and chemical modification of GO are not affected by the simultaneous reduction of Ag and the deposition of AgNPs on the graphene surface. Above 600 °C, however, a slight increase in the burning temperature of *f*-rGO in the presence of AgNPs is observed similarly to other works, possibly due to the higher thermal stability of the *f*-rGO/AgNPs hybrid [51]. In the presence of Ag, the reduction of GO is catalyzed by Ag nanoparticles, resulting in a more graphitic *f*-rGO/AgNPs product, and therefore, more thermal stability. This is also confirmed by the red-shifted λ_max_ in the UV-Vis spectrum of the *f*-rGO/AgNPs hybrid (at 280 nm, Figure 4) compared to that of the *f*-rGO (at 270 nm, Figure 4), and by the more intense peak in the X-ray diagram corresponding to the graphitic nature of the *f*-rGO/AgNPs at 25 degrees (Figure 5).

### 3.4. Raman Analysis of f-rGO/AgNPs

**Figure 7:**
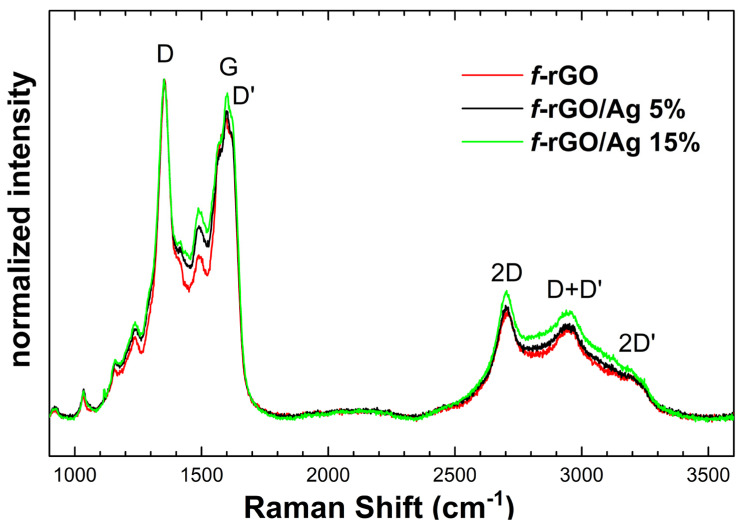
Raman spectra of rGO and *f*-rGO/AgNPs samples. All spectra are the average signal from a mapping at 25 points.

Raman analysis (Figure 7) has shown the main graphitic fingerprint features of *f*-rGO, i.e., the D and G bands at 1352 and 1598 cm^−1^, respectively, as well as at higher frequencies the second order 2D, D+D’ and 2D’ bands at 2702, 2943 and 3217 cm^−1^, respectively. The D band is attributed to the break of sixfold aromatic lattice rings due to the existence of defects since this vibration is excluded for a perfect lattice structure. Thus, the D peak (integrated intensity) can be considered as a measure of the lattice deviation from perfection. Its sp^3^ C–C vibration type is assigned to an A_1g_ (sometimes also referred to as breathing) mode at the Brillouin zone boundary. The G band is attributed to the in-plane stretching vibration motion of the C=C bond which is assigned to E_2g_ mode at the Brillouin zone center Γ [52]. This vibration is a sp^2^ doubly hybridized in a perfect graphitic lattice, but the presence of impurities or damaged bonds raises the degeneracy. This is illustrated in Figure 7’s spectra where the “G peak” consists of a triplet of peaks. The two satellite peaks that seem to be red- and blue-shifted by a few wavenumbers (cm^−1^) on both sides of the central and stronger G peak frequency are not originating from stress effects. The band at about 1620 cm^−1^ has the same origin as the D band: the presence of defects. The band at 1570 cm^−1^ can be attributed to carbon bond vibrational coupling deviations in the vicinity of the sp^3^ defect site due to the reduction and functionalization processes [53,54].

At higher frequencies, the D+D’ combination band requires a defect for its activation, while no defects are required for the activation of 2D and 2D’ second-order overtones [55]. Moreover, along Raman spectra, there are several bands with lower intensity that can be attributed either to graphitic structural defects or to impurities. Finally, although the I_D_/I_G_ ratio cannot be accurately determined, its value is practically independent of Ag concentration. This means that AgNPs do not have any contribution to lattice deterioration.

In conclusion, the spectra of *f*-rGO/AgNPs samples were quite similar to that of *f*-rGO, indicating that the one-pot synthesis of the hybrid has no effect on the structure of the final *f*-rGO/AgNPs.

### 3.5. Ink Formulation and Electrical Properties of f-rGO/AgNPs Pigment and Inks

As already described, the stable water-based inks were prepared by mixing the *f*-rGO/AgNPs hybrid with a mixture of three selected resin emulsions (acrylic and styrene-acrylic emulsions). However, it has been established by a series of experiments (not presented in this work) that the resins system can be based either on an acrylate emulsion, or a styrene acrylate ester emulsion, polyurethane, polyurethane-polyacrylate (or their salts) or a combination of the above (also with other resins). The high hydrophilicity of the conductive pigment favors its dispersion in the resin emulsions (and thus its printability) without requiring the time-consuming basic three steps (Premix-Grinding-Let-down) applied in industry for the incorporation-dispersion of the pigments in the resin system to form the inks. It was observed that the graphene hybrid can be easily mixed and homogenized with commercial resins for the preparation of the conductive graphene ink systems using special laboratory or industrial homogenizers for 5 min with excellent results.

**Figure 8:**
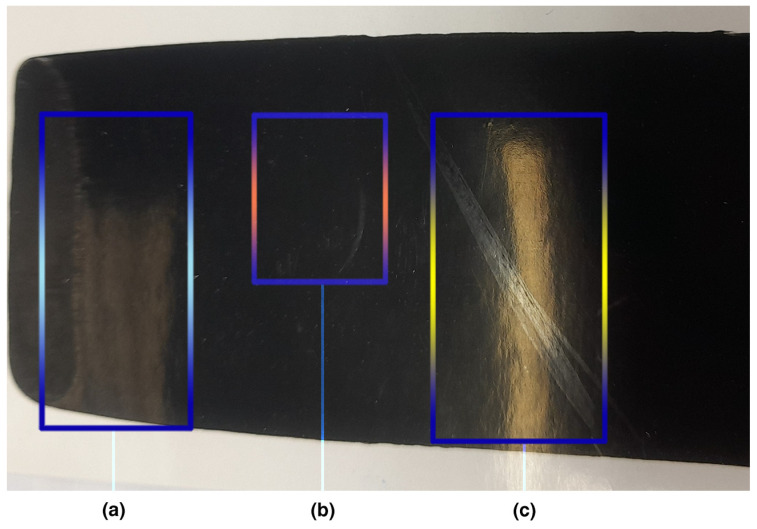
Representative examples from the hybrid ink (**a**) adhesion, (**b**) water rub resistance and (**c**) scratch resistance test.

Although several other resin mixtures have been successfully tested, the more environmentally friendly styrene-acrylic emulsion Joncryl MB90 (instead of Joncryl 90) was selected because this emulsion is a biomass balance product produced by renewable raw materials and can thus improve the carbon footprint of the process without reducing the print quality. It was found that the as-prepared stable water-based ink needs only a few minutes of shaking before use and does not require any special treatment (e.g., heat treatment or the use of pulsed light) for the improvement of its conductivity. Concerning the printed ink properties, the absence of carbon black or copper phthalocyanine or graphene ink residues on the pressure-sensitive tape denotes the lack of adhesion problems (Figure 8a). Also, the examined printed samples did not show any redissolution of the conductive ink from the tested area after its contact with water (Figure 8b). The water rub resistance represents the high level of graphene pigment entrapment into the system of resins (Figure 8b). Similarly, the results from the scratch resistance test were positive (Figure 8c). Finally, the heat resistance test did not show any visible transfer of graphene ink from the face of the printed surface to the aluminum foil.

To examine the electric conductivity of the *f*-rGO/AgNPs pigment and *f*-rGO/AgNPs ink, circular spots (~400 μg dispersed in 50 μL οf water) were formed by drop casting on paper and then air dried. The values of the sheet resistance (R_s_) were 4.3 Ohm sq^−1^ for the *f*-rGO/AgNPs, ~9 Ohm sq^−1^ for the Gravure/Flexo Ink 55-45 and ~6 Ohm sq^−1^ for the Screen Ink 70-30 (Table 1). Ιnks prepared by the same resins and the two components (*f*-rGO and AgNPs) separated at similar percentages (e.g., 50.3% carbon or 4.5% Ag) showed a much higher resistance indicating an important synergistic effect between the two components.

### 3.6. Rheology of Inks

**Figure 9:**
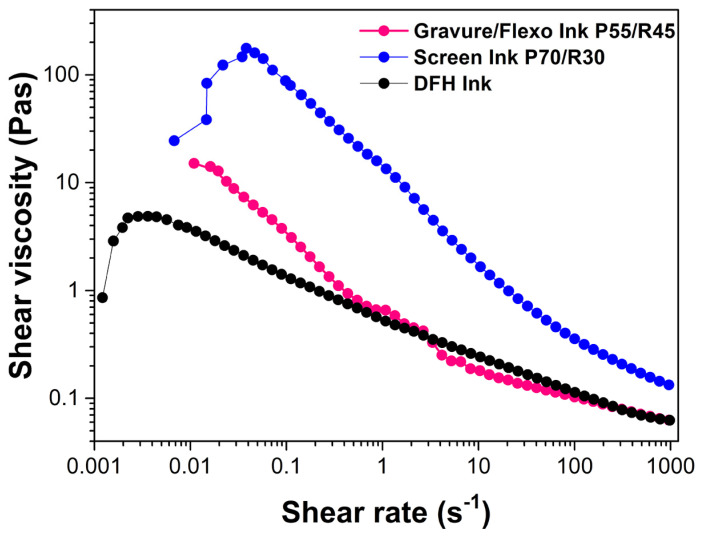
Viscosity of Gravure/Flexo Ink P55/R45, Screen Ink P70/R30 and a commercial nonconductive flexo ink from Druckfarben Group as a function of shear rate with the ratio of pigment solids/resin solids as a parameter.

The two printing inks, Gravure/Flexo Ink P55/R45 and Screen Ink P70/R30 were formulated using the same *f*-rGO/AgNPs pigment. Their viscosities were adjusted to be printable for gravure, flexography and screen-printing methods and were found to exhibit shear-thinning properties (Figure 9). The excellent dispersibility of the hybrid pigment allowed the preparation of conductive inks of high viscosity (up to 50 Pas for gravure and flexography, 100–150 Pas for screen-printing) under low shear rates, which are much higher compared to other conductive inks. However, in high shear rate ranges (according to the operation conditions of printing machines) the ink viscosity is in the usual range of 0.05–0.3 Pas and thus the inks are efficient during printing. Specifically, when the mass of the conductive *f*-rGO hybrid doped with metal nanoparticles is 5–25% with respect to the total mass of the ink then the ink viscosity, under high shear rates, is in the range of 0.05–0.3 Pas so that it is suitable for gravure/flexography. Whereas, when the mass of the conductive hybrid pigment to the total mass of the ink is 10–35%, then the ink viscosity, under high shear rates, is in the range of 0.1–0.5 Pas and the ink is suitable for screen-printing.

The appearance of shear thickening behavior at very low rates of shear has been observed by several researchers for many polymeric systems. It can be attributed to higher solids concentration [56,57] or it can be an artifact due to the non-equilibration of the sample [58].

### 3.7. Printing of Conductive Inks and Electrical Characterization of Their Printed Patterns

**Figure 10:**
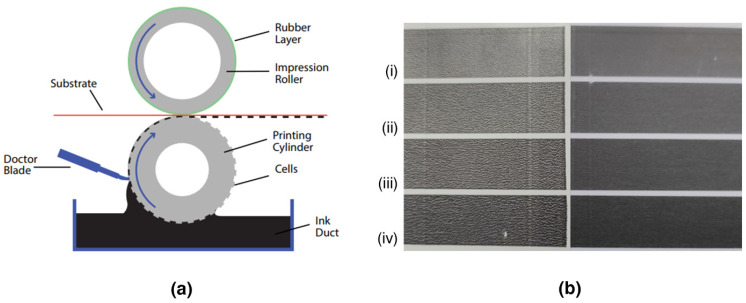
(**a**) Illustration of the principle of roll-to-roll gravure printing (**b**) Gravure-printed items on a standard IGT coated paper (C2846, 150 g m^−2^) (left) and on an extra premium high-glossy photo paper (265 g m^−2^, @work) (right) with the *f*-rGO/AgNPs ink with total solids pigment/resin ratio 55/45 (Gravure/Flexo Ink P55/R45). The printing force between the engraved disc and the substrate was 200 N and the printing speed was 0.6 m/s. The thickness of the ink layer increases from i to iv.

**Figure 11:**
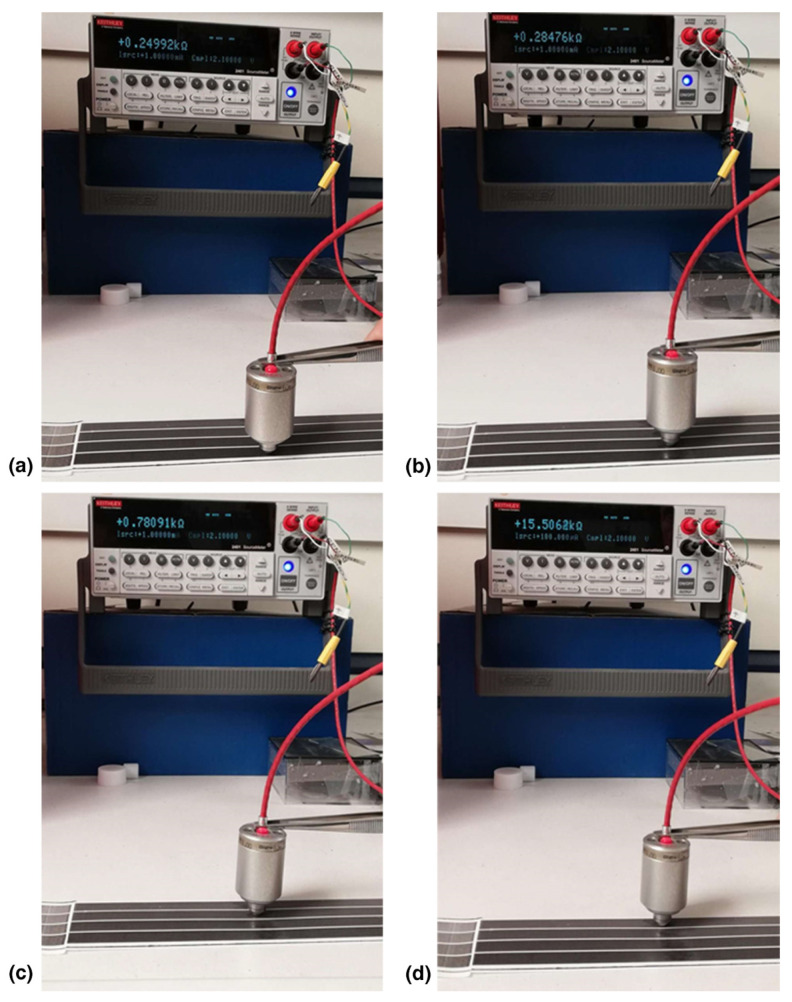
Measurements of the V/I of each of the four zones of different thickness ink layers using an extra premium high glossy photo paper and the Gravure/Flexo Ink P55/R45. The measurement of V/I increases from photo (**a**–**d**) as the thickness of the ink layer of each zone reduces.

**Figure 12:**
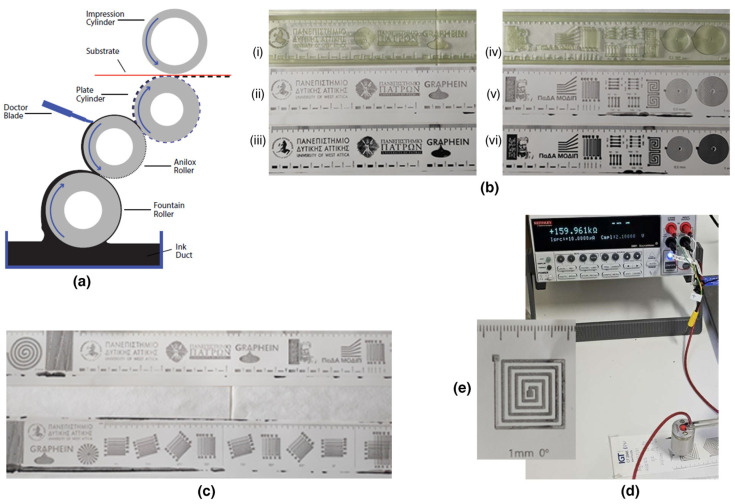
(**a**) Illustration of the principle of flexographic printing (**b**) **i**,**iv**: The as-used photopolymer plate Torelief WF80DHX4 (thickness 0.80 mm, 65 Shore D hardness); **ii**,**v**: Photos of different printed patterns on the same paper (CT2846, IGT Testing Systems) using the Gravure/Flexo Ink P55/R45. Anilox/printing force 50/50 N, anilox/printing speed 0.5 m s^−1^ and doctor blade pressure 6 N; **iii**,**vi**: Comparison with a commercial nonconductive flexo ink of Druckfarben Group. (**c**) Printed patterns using the photopolymer plate WF80DHX4 (up) and DPN 67 (down). (**d**,**e**) Measurement of resistance (V/I) of a printed line with width 1 mm using the Gravure/Flexo Ink P55/R45.

**Figure 13:**
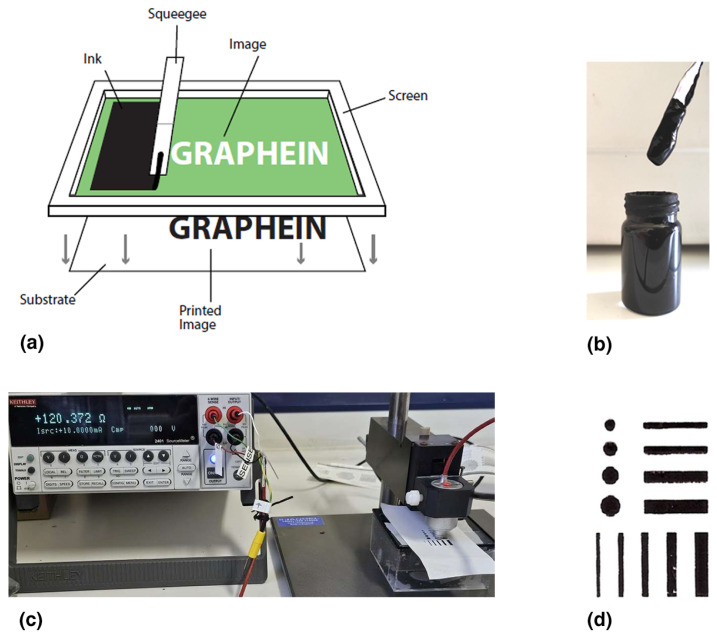
(**a**) Illustration of the principle of screen printing (**b**) Photo of the Screen Ink P70/R30 (**c**) Measurement of the resistance of a printed dot of the screen-printed pattern (**d**) on an extra premium photo paper.

The as-prepared stable and high-performance inks with tailored rheological properties were applied with three different printing methods (i.e., gravure, flexography, screen printing), aiming for acceptable substrate–ink interaction using different kinds of low-cost coated papers (Figure 10, Figure 11, Figure 12 and Figure 13). The ink transfer is an extremely complex nonlinear phenomenon affected by various parameters that hinder the control of the process [59,60]. However, the exhaustive study of the effect of these parameters was not within the scope of this study.

#### 3.7.1. Gravure Printing Tests

Gravure predominates mainly in the high-volume printing of packaging, wallpaper, gift wrap, magazines, advertising, etc., while offering a powerful approach to the fabrication of printed electronics in high volume and at the highest speed [61]. A gravure printing unit includes the engraved gravure cylinder, the ink-circulation system, the adjustable doctor blade and the impression roller (Figure 10a). The process comprises an image engraving onto the surface of a gravure metal printing cylinder in the form of cells acting as ink wells. The cylinder is dipped in the ink, filling the cells that have different depths. The excess of ink is removed from the non-image areas by doctor blades. The cells that hold the ink transfer it directly to the substrate as this is pressed and rolled between the printing and the impression cylinders [62].

Table 2 summarizes the R_s_ values in kΩ sq^−1^ for four zones of different thicknesses of the ink layer when the Gravure/Flexo Ink P55/R45 and a special gravure cylinder were used. In accordance with our previous studies [30,31,44] it was observed that the more the thickness of the printed zones the less the R_s_ values (Figure 10 and Figure 11, Table 2). Table 2 also includes the R_s_ values of other similar conductive inks, for the same four zones, after printing under the same conditions including the same printing cylinder and the same equipment [30,31,44]. This comparison shows that although the percentage of Ag is less compared to other *f*-rGO/AgNPs hybrids prepared using different synthesis methods, the current method leads to printed patterns where lower R_s_ values (and thus enhanced conductivity) were achieved. It is clear that the dispersion of AgNPs on the *f*-rGO creates conductive interconnections between the graphene nanosheets, improving the electrical conductivity of the final pigments [31]. Similar, to our previous study [31], it was found that the best interaction between the ink and the substrate, and thus the best printing quality, was obtained with a common high glossy photo paper (Figure 10b). Probably the low surface roughness of this smooth paper contributes to a lower contact angle compared with the IGT standard coated paper (Figure 10b).

#### 3.7.2. Flexography Printing Tests

Flexography is a roll-to-roll printing method using flexible relief printing plates that are engraved rubber or photopolymers image carriers (flexographic plates) (Figure 12a). The ink is transferred from the ink-circulation system via an engraved roller (anilox) onto the flexographic plate where any excess of the ink is removed by the doctor blade and then it is directly printed on the substrate passing through the impression cylinder and the image carrier that is placed on the flexo plate cylinder. Although almost all types of substrates can be printed with this method, it is widely used in flexible packaging, labels, plastic bags and sacks. Also, flexo is used for printing on folding cartons and corrugated cardboard. The popularity of flexography is increasing, e.g., in the medical/pharmaceutical industries, and in publishing, while recently it has attracted the interest of researchers in the field of functional printing [63,64,65,66].

As in the case of the gravure printing tests, satisfying printing results were obtained when the low viscosity *f*-rGO/AgNPs ink formulation was tested for flexography (Figure 12b–e). In accordance with previous results based on the flexographic printing of a highly conductive and dispersible graphene/multiwall carbon nanotube hybrid [63], it was found that the pre-inking process of the plate before printing was crucial and favorable for the print quality while the effect of printing pressure and printing speed do not have the same definite effect. Furthermore, promising printing results were noted when using flexographic printing plates with a hardness of 65 Shore D and 67 Shore A despite the fact that their thickness was different (Figure 12c). Acceptable print results were obtained using the CT2846 paper of the IGT Testing Systems (while up to now we have observed similar printing results when using substrates of sufficient smoothness as the smart paper type-2 [45] or photo paper of premium quality). Proper wetting of the conductive ink on the printing plate’s surface was one of the important parameters considered for the correct selection of the photopolymer material(s) used in this work. In the next stage, the investigation of this critical parameter could give even better print results and more tailored surface properties.

As expected, the R_s_ values of the flexo-printed patterns were higher than those of the gravure ones due to their lower thickness [64]. Generally, the dry ink-film weights applied in the gravure and flexographic processes are between 1.5 and 3 g m^−2^ which corresponds to a wet film weight of 5–9 g m^−2^ for gravure and 3–6 g m^−2^ for flexographic processes [67]. Specifically, the R_s_ values were less than 725 kOhm sq^−1^ determined by the printed structure (e.g., the line width) (Figure 12e). However, there is room for improving the printing results and thus the R_s_ values using printing plates with optimized technical characteristics and printing with optimal conditions. Parameters such as the type of the photopolymer printing plate, its hardness and surface properties like surface free energy, its production method and ultraviolet radiation post-treatment, the pre-inking process, etc., are essential for the successful transfer of the ink from the printing plate to the substrate and the improvement of the qualitative properties of the printed patterns [68]. The optimization of such parameters was not among the main goals of this work and for this reason, they were not investigated in greater depth. However, R_s_ values can, in general, be improved by applying multilayer and/or hybrid printing [59,65].

#### 3.7.3. Screen Printing Tests

Screen printing is an important method for fine arts, for the printing of fabrics and textiles, the fabrication of solar cells [59,64,69,70] as well as for the electronics industry. It is noted that traditional printed-circuit boards have been produced in industry for the last sixty years with flatbed screen printing [70]. Nowadays, this mature technology is expected to dominate in the production of flexible printed electronics while roll-to-roll rotary screen printing can be fully adapted [59,70]. To print with flatbed screen printing, a squeegee and an emulsion screen mask are used. The emulsion screen mask is composed of a plate frame (usually aluminum) and a mesh (stainless steel, polyester or other specialized ones) coated with a photosensitive emulsion. The mesh having the designed pattern is tightly stretched on the frame (Figure 13). The pores of the mesh are blocked up in the non-imaging areas by the photosensitive emulsion while the remaining pores in the imaged areas are left open to allow ink to pass through. The ink is placed on the screen mask and the substrate is placed under the screen. The squeegee presses (manually or automatically) the ink to flow through the open pores of the mesh as it is drawn across the screen. Thus, the designed pattern is formed on the substrate [64,70].

As already mentioned, a simple manual mechanism was used for the screen-printing preliminary tests. The ink produced for gravure and flexography was also suitable for screen-printing when the total solids pigment/resin ratio was increased from 55/45 to 70/30. The high dispersibility of the *f*-rGO/AgNPs hybrid permits reaching a high pigment load. This is a very advantageous result since there is currently a lack (to the best of our knowledge) of conductive inks with properties suitable for all three methods (gravure, flexography, screen printing). A typical photograph of the screen-printed pattern that was easily fabricated using the *f*-rGO/AgNPs nanohybrid developed in this work is shown in Figure 13. Also, it was found that the R_s_ of the screen-printed patterns were below 567 Ohm sq^−1^, mainly due to the thicker screen-printed layer produced compared to flexography and gravure [64].

## 4. Conclusions

A new one-step aqueous synthesis of highly conductive (and dispersible in various polar solvents) *f*-rGO/AgNPs hybrids using 2,5-DBSA as a reducing agent was presented in this work. The aminophenylsulfonic group-containing reducing agents can provide modified, arylsulfonate-containing graphene materials with a high hydrophilicity which contributes to an increased conductivity due to the formation of a continuous conductive network through the pigment sheets.

Most importantly, the *f*-rGO/AgNPs hybrid allowed the development of highly conductive water-based printing inks that (a) can be formulated by using resins that are already used in the printing industry and (b) fulfill the requirements of three different printing methods (gravure, flexography and screen printing). For the as-prepared inks, no additives, surfactants, rheology modifiers, anti-foaming agents, thickeners, plasticizers or solvents were used for the respective formulations and no post-printing treatment stages (e.g., thermal, chemical, electrical, photonic or plasma sintering) were required. Finally, the successful choice of paper substrates, the very good adhesion and resistance to heat, scratch, and water rub were very important factors that contributed to the obtained high printing quality. Also, the successful development of a conductive ink with increased solid content and thus a high load in conductive material without agglomeration is clearly an additional advantage.

In a future communication, we will report the results of our work on the scalability of the synthesis process that has been made possible after surpassing the slow and copious synthesis steps. These are not connected with the actual reduction procedure but rather the time-consuming stages involving the filtration and purification/washing of the products.

## 5. Patents

Water-based conductive printing ink with a pigment produced by one-step reaction, international application No. PCT/EP2023/068591 based on the National (Greek) protection title 1010492, National (Greek) application No. 20220100543, 07 July 2022.

## Figures and Tables

**Table 1:** Composition and sheet resistance, R_s_, values of the two conductive inks for gravure, flexo and screen printing.

Ink	Total Solids Pigment/Resin Ratio	Pigment Solids/Ink Solids	Resin Solids/Ink Solids	Total Solids in Ink (%)	Pigment Solids in Ink (%)	R_S_ (Ohm sq^−1^)
**Gravure/Flexo Ink P55/R45**	55/45	0.55	0.45	21.8	13.8	9
**Screen Ink P70/R30**	70/30	0.70	0.30	29.5	23.4	6

**Table 2:** The R_s_ values of various *f*-rGO/AgNP inks derived from stripes of various thicknesses (EP: extra premium paper, PP: photo paper, HG: high glossy paper).

***f*-rGO/AgNPs Inks**	**%Ag** **(*w*/*w*)**	**Reducing Agent**	**Ratio of Pigment Solids/** **Resin Solids**	**Cell Volumes (mL m^−2^)**				**Paper Type**	**Ref.**
	**Kind of Nano** **Materials**			**16**	**11**	**9**	**7**		
				**R_s_ (kOhm sq^−1^)**					
	8.5	2,5-DBSA	55/45	1.1	1.3	3.5	70.2	EP-PP-HG	This work
	Nano particles								
	10	2,4-DBSA	50/50	1.5	6.8	9.0	17.0	C2846	[30]
	Nano wires		70/30	8.0	3.5	7.5	16.3		
	18	2,4-DBSA/ DMF	50/50	9.6	27.3	118	4500	PP-HG	[31]
	Nano particles								

## Data Availability

The data presented in this study are available on request from the corresponding authors.
